# Blood Gene Expression Network Expression Strongly Relates to Brain Amyloid Burden

**DOI:** 10.1002/alz.095089

**Published:** 2025-01-09

**Authors:** Vaibhav A Janve, Mabel Seto, Reisa A Sperling, Paul S. Aisen, Robert A. Rissman, Mary Ellen I. Koran, Logan C. Dumitrescu, Rachel F Buckley, Timothy J. Hohman, A4 and LEARN Study Group

**Affiliations:** ^1^ Vanderbilt Memory & Alzheimer’s Center, Vanderbilt University Medical Center, Nashville, TN USA; ^2^ Vanderbilt Genetics Institute, Vanderbilt University Medical Center, Nashville, TN USA; ^3^ Center for Alzheimer Research and Treatment, Brigham and Women’s Hospital, Harvard Medical School, Boston, MA USA; ^4^ Department of Neurology, Massachusetts General Hospital, Harvard Medical School, Boston, MA USA; ^5^ Center for Alzheimer’s Research and Treatment, Brigham and Women’s Hospital, Massachusetts General Hospital, Harvard Medical School, Boston, MA USA; ^6^ Alzheimer’s Therapeutic Research Institute, Keck School of Medicine, University of Southern California, San Diego, CA USA; ^7^ Department of Radiology, Vanderbilt University Medical Center, Nashville, TN USA; ^8^ Melbourne School of Psychological Sciences, University of Melbourne, Melbourne, VIC Australia; ^9^ Alzheimer’s Clinical Trial Consortium, Boston, MA USA

## Abstract

**Background:**

Amyloid deposition occurs during the preclinical stages of Alzheimer’s disease (AD) a decade or more before clinical symptoms emerge. We leveraged blood transcriptomics and positron emission tomography (PET) measures of amyloidosis to identify cell types and gene networks in the blood that relate to amyloid burden in the brain.

**Method:**

Whole blood RNA sequencing and amyloid PET data were leveraged from 1771 participants (62% females, mean age 71, 32% amyloid+) in the Anti‐Amyloid Treatment in Asymptomatic Alzheimer’s Disease (A4) Study. RNAseq data were aligned with STAR, sorted, counted using Subread, and batch normalized with voom. 19,945 genes were available for analysis following quality control. Cell fractions were quantified using CIBERSORTx (LM22 used as reference) and WGCNA was used to calculate gene co‐expression modules (soft threshold = 12). Amyloid PET data were acquired using 18F‐florbetapir 50‐70 minutes post injection and a global cortical standardized uptake value ratio (SUVR) was quantified (whole cerebellum as reference). Linear regression related gene module expression or cell fraction to mean SUVR, covarying for age, sex, education, *APOE ε2* and *ε4* status. Correction for multiple comparisons used false discovery rate. Gene enrichment was performed using Gene Ontology.

**Result:**

Two gene modules were associated with amyloid deposition. One included 48 genes whereby higher expression was associated with lower amyloid burden (β = ‐12.37, p = 0.0001). This module was enriched for DNA damage pathway genes and the top gene associations in the module included histone genes such as *H3C3* and *H2AC14*. The second module included 241 genes whereby higher expression related to higher amyloid burden (β = 7.15, p = 0.001). This module was enriched for leukocyte migration and regulation of RNA pathways. The top gene association in the module was the calcium homeostasis gene *CHERP*. Finally, a higher proportion of CD4 positive activated memory T cells was associated with higher amyloid burden (β = ‐1.940, p = 0.04), but did not survive FDR correction.

**Conclusion:**

Our results implicate DNA damage and activated memory T cells as contributing to amyloid deposition in preclinical AD. Future work will seek to identify modifiers of the *APOE* effect on amyloid and evaluate blood transcription networks as complementary biomarkers of amyloid deposition.

